# The Extraction of Teeth

**Published:** 1861-04

**Authors:** 


**Extract from the discussion of the New York Dental Society on the Extraction of Teeth.**

The Extraction of Teeth.—Dr. Allen could not hope to say anything new on the subject; but he would give his mode of practice. He would commence with the extraction of superior incisor teeth. He used a small-beaked forceps and slit the gum from the outer and inner surface, making a perpendicular cut a little above, especially if the crown of the tooth be so decayed that there is any danger of its breaking. The instrument would then pass up on the neck of the tooth, firmly embracing it, and by using a rotary instead of a lateral motion the tooth can then be very easily started from the socket.

He used but very little lateral motion in removing a tooth, especially incisors or canine teeth. The rotary motion will take it out without fracturing the alveolar, and the amount of force required is far less than from lateral motion. If it is a fang of a tooth, he used the Parmlee forceps, with a sharp-edge beak, which he passed up, separating the gum, and embracing a portion of the alveolar process, and the fang will usually come out very readily. By the same movement he removed canine teeth with equal success. He was aware that the screw forceps had been used, but in his own practice he never found it of any great value. Using the screw with a deep incisor, he found it very difficult to get the thread in sufficiently firm to remove the fang; if an inflamed tooth, the force necessary to work the screw in would be so great as to give unnecessary pain. He seldom used the screw at all, but depended upon the forceps and elevator almost entirely. The old-fashioned turn-key he had not used for many years, and he found no case where it became necessary to do so; and although he found some worthy dentists, with whom he had been acquainted for many years, occasionally using it, he thought it was more a mattar of habit than any thing else. He did not use the Parmlee forceps for incisor teeth in every instance; there was one with a very thin beak he sometimes used; but for bicuspids he used the Parmlee more than the other; and in extracting bicuspids, he would remark that he employed the same rotary motion that he did in extracting incisor and canine teeth, succeeding almost invariably without using sufficient lateral force to fracture the alveolar process. For the fangs of bicuspids and incisors, he used a small-pointed forceps which he could pass far up on the fang With these three instruments he was able to take out all the incisor, canine, and bicuspid teeth in the upper jaw. For the upper molars upon the right side he used a forceps with a beak that would embrace the inner portion of the tooth and a point passing between the two outer fangs of the two molars. For the left side, he used an instrument just the reverse of the other in shape—the ordinary right and left molar forceps. He made it a point to embrace a molar tooth above the crown, by crowding it up until it passed between the fangs, and by embracing the inner fang, he seldom failed to make a successful operation. If the crown be decayed, he used one of the smaller beaks and took out the fangs separately. If he found the fangs still connected and the crown all gone, he used a sharp instrument, like an elevator, separated the fangs, and, with a small-beaked forceps, picked the parts out. In extracting the *dens sapientia*, or wisdom teeth, if entirely behind the other teeth, he used a bayonet shaped forceps invariably, although he had an instrument, highly recommended, which came together like a cutting instrument, forcing the tooth out.

In reply to an inquiry from the Chair, as to whether he was in the habit of lancing gums,

Dr. Allen said, if the gums adhered to the teeth he thought it important to lance them, but when the gums were in a spongy condition he seldom used the lancet. He preferred not to use the lancet when not necessary, for the reason that it frequently frightened the patient as much as the extracting instrument. Still he knew instances in which serious injury was done by not using the lancet. One case in Cincinnati, he recollected, where a dentist, in attempting to extract a wisdom tooth for a lady without lancing, tore the roof of the mouth so badly that it had to be stitched up, which could not have occurred had the gum been properly lanced. Therefore it was necessary to use the lancet in most instances; and an experienced dentist would be always able to judge when to do so. Where he had a number of teeth to extract for one person, and found the gums in a condition that they adhered to the teeth, he often separated half a dozen at a time, then took his forceps and extracted four, five, or six at a sitting, in quick succession. Instead of stopping every time, and letting the patient spit out the blood, he did not find it necessary to stop more than two or three times in pulling out eight or ten teeth. This method he approved of strongly, for he found that the quicker an operation of this kind was over the better. Frequently he had patients who would come in and say, "I want one tooth taken out to-day, and another to-morrow, and next week another,—but I can't stand more than one at a time." He generally succeeded, however, in getting them to have all taken out before leaving the chair. In the extraction of the lower teeth, he used for the incisors a small-beaked hawk-bill forceps, and for the bicuspids below, he used a hawk-bill—the ordinary left and right forceps, and for the *dens sapientia*, in the inferior jaw, he also used the hawk-bill forceps, and no other. For removing fangs in the lower jaw, he frequently used an elevator. Upon the right side he used a kind of punch, and, slitting the gum, threw his weight upon the punch, resting his arm against his body, which should be always done in using an elevator; otherwise, if the fang should crumble, there would be danger of the instrument going too far and injuring the tongue or mouth; but by having the forearm rest against the body, there was only the play from the muscle of the arm, and no danger of the instrument doing injury. It required some dexterity to use an elevator properly, but he regarded it with favor. In some cases he used the hook elevator, with which he removed a tooth or fang very easily. These were about all the instruments he used in extracting teeth.—*New York Dental Journal.*

---

PHOSPHORNECROSIS has become prevalent among the makers of lucifer matches in France. The Academy, at the solicitation of the Government, recommends, as a means of prevention, that matches be made of pure amorphous phosphorus.—*Baltimore Journal of Medicine.*

HYDRATED SESQUIOXIDE OF IRON AS AN ANTIDOTE TO POISONING BY ARSENIOUS ACID, *by Wm. Watt, M. D.*—The hydrated sesquioxide of iron was proposed as an antidote for poisoning by arsenious acid in 1834, by Drs. Bunsen and Berthold, of Gottingen. These gentlemen discovered that if a solution of arsenious acid was agitated with a quantity of the hydrated sesquioxide of iron, that the former was precipitated in a very insoluble form. A knowledge of this fact induced them to make a number of experiments upon animals, in order to test its antidotal powers. Of the experiments they made favorable statements, which were afterward confirmed by the experiments of Orfila, Lesueur, Soubeiran, and others. Opposed to the evidence of these experimentalists, we have only the unfavorable results of Mr. Brett and those of Mr. Orton. It was afterward ascertained, however, that the amount of the antidote used by these gentlemen was uniformly too small. More recently Fasoli has made some experiments on dogs with the hydrated oxide. He states that having procured nineteen dogs, he administered arsenious acid to five of them without the antidote; they all died. To the others, arsenious acid being administered, was followed by the antidote, and they all recovered. Unfortunately, he does not state the amount of arsenic or antidote used in any of his cases. We have a number of cases recorded, in which it is claimed that this antidote has been the means of saving life in the human subject. However, in a majority of the cases it was not known that the amount of arsenious acid taken would have been sufficient to have produced death.

When used as an antidote, it should be administered in its recently precipitated gelatinous condition, as it appears to lose its neutralizing powers in proportion to the time it is kept. It is thought advisable, in order to insure its antidotal effects, to administer about twelve parts of the antidote to one of arsenious acid. But as the antidote is incapable of producing any injurious effects, it may be administered in large quantities. According to Graham, the mutual reaction of the hydrated sesquioxide and the arsenious acid gives rise to the formation of the arseniate of the protoxide of iron. This change would be represented by the following formula:

$$2\,Fe_2O_3 + As\,O_3 = 4\,Fe\,O + As\,O_5$$

Taylor says that the hydrate is incapable of acting as an antidote when the arsenious acid has been taken into the stomach in the form of powder. The hydrated oxide, in order to act as an antidote to poisoning by the arsenic salts, requires to be combined with an acid, which may separate the base; then the arsenious acid and the oxide react on each other.

The above results being somewhat discrepant, we, at the suggestion of Professor Wormley, undertook a series of experiments in his laboratory, for the purpose of satisfying ourselves of the real value of the antidote in poisoning by arsenious acid when administered to dogs. We were kindly assisted in the experiments by Dr. Willis and Mr. Pinnell.

The antidote was prepared by precipitating the muriated tincture of iron of the shops with ammonia, filtering through muslin, and washing the filter until the washings were free from ammonia. The mass was then drained.

In administering the antidote about two table spoonsfull of the magma were given to each dog.

Before making any experiments as to the action of the antidote, it was thought advisable to ascertain the effect of the arsenious acid solution alone upon the animals. The first seven cases were, therefore, confined to the acid solution without the antidote.

In the first fifteen cases, the arsenious acid was in solution in the proportion of two grains of the acid in one fluid ounce of water.

*Experiment* 1*st; weight of dog* 50 *lbs.*—Two grains of arsenious acid administered; symptoms of pain came on in fifteen minutes; which were followed in ten minutes by vomiting, which continued about four or five hours, and were then followed by bloody purging. From this time the animal recovered.

*Experiment* 2*d; weight* 60 *lbs.*—Three grains administered; symptoms of pain came on in twenty minutes, and vomiting in about thirty minutes. The vomiting continued with great severity for about six hours; it then gradually ceased, and was followed by purging of blood, which continued about two days, during which time the animal refused to take any food. He finally recovered.

*Experiment* 3*d; weight* 18 *lbs.*—Three grains administered; symptoms came on in fifteen minutes; the animal apparently suffered great pain, which he manifested by frequently changing his position, whining, &c. He did not vomit for about thirty minutes after the poison had been administered. The vomiting was continued and severe. The matters ejected consisted of a thick white frothy tenacious mucus, which the animal had great difficulty in expelling from the mouth. The vomiting ceased, and he gradually lost the use of his limbs, and died cômatose, six hours after the poison had been administered.

*Experiment 4th; weight* 15 *lbs.*—Three grains administered; symptoms came on in fifteen minutes, followed in five minutes by vomiting, which continued about five hours. The animal died in eight hours after the administration of the arsenic.

*Experiment 5th; weight* 12 *lbs.*—Six grains administered, but part of the solution evidently passed into the trachea, as its administration was followed by strangulaticn, coughing, difficult breathing, etc.; vomiting came on almost immediately, and continued about an hour and a half, when the animal died.

*Experiment 6th; weight* 25 *lbs.*—Six grains administered. Symptoms of poisoning came on in eight minutes, followed immediately by vomiting and symptoms of pain. The vomiting which was very severe, at first consisted of a thick glairy mucus, but soon became mixed with a considerable amount of arterial blood. The animal died in about six hours.

*Experiment 7th; weight* 30 *lbs.*—Six grains administered. In about three minutes the animal showed evident signs of pain, and vomited in five minutes. The vomiting was severe, and continued almost without interruption for five hours, when it ceased, and the animal becoming comatose, died.

*Experiment 8th; young dog, weight* 10 *lbs.*—Two grains administered, followed in about five minutes by the antidote. The animal showed some signs of the action of the poison in about forty minutes, but did not vomit for about an hour; he then vomited once, after which he became stupid and was inclined to sleep. These symptoms continued for about three hours, after which he appeared perfectly well.

*Experiment 9th; weight* 15 *lbs.*—Three grains administered, followed immediately by the antidote. Symptoms of pain came on in thirty minutes, and vomiting in about one hour after the poison had been administered. The vomited matter consisted of the antidote mixed with the alimentary matter of the stomach. This animal recovered.

*Experiment* 10*th; weight* 12 *lbs.*—Three grains administered This was followed in about ten minutes by the antidote. Symptoms of poisoning came on in thirty minutes after the arsenic was administered. He then showed some signs of pain, and vomited two or three times—the vomited matter consisted of mucus mixed with the antidote. During the first three hours after the appearance of the symptoms, he was somewhat stupid, but at the expiration of that time he appeared about as well as usual.

*Experiment* 11*th; weight* 35 *lbs.*—Four grains administered, followed immediately by the antidote. The animal showed no symptoms for upwards of an hour; he then became slightly uneasy, being inclined to move about. These symptoms, though slight, continued for an hour when he vomited twice; the vomiting was attended with but slight effort and was not repeated. Symptoms abated, and the animal appeared to have recovered perfectly in three hours after the arsenic was administered.

*Experiment* 12*th; weight* 20 *lbs.*—Four grains administered and followed immediately by the antidote. The animal showed no symptoms for more than an hour; he then appeared to be suffering from pain. One hour and a half after the administration of the poison he vomited; the vomited matter consisted of the antidote mixed with a small amount of mucus. The vomiting was not repeated. This animal recovered speedily, partaking of food in three hours after the poison had been administered.

*Experiment* 13*th; weight* 40 *lbs.*—Five grains administered. The antidote was not administered until ten minutes after the arsenic. The animal showed marked symptoms of pain in twenty minutes, then vomited a considerable amount of a thick glairy mucus mixed with the antidote; made several attempts to vomit afterward, and appeared somewhat dull and stupid for about six hours. The symptoms gradually diminished and he recovered in about eight hours.

*Experiment* 14*th; weight* 18 *lbs.*—Six grains administered, followed immediately by the antidote; symptoms of poisoning appeared in ten minutes, vomited in fifteen, and appeared to suffer some pain for about six hours, during which time he made a number of attempts to vomit. This animal showed none of the symptoms of depression or stupor that marked some of the other cases. The animal recovered in about eight or nine hours.

*Experiment* 15*th; weight* 15 *lbs.*—Six grains administered and followed in about five minutes by the antidote. The animal vomited almost immediately after the antidote was administered. The vomited matter consisted of a large amount of greased paper which the animal had eaten; with this was mixed the entire amount of antidote administered, as was shown by the subsequent vomiting. The antidote was not repeated. The symptoms gradually increased in severity, and the animal died in about six hours from the time the arsenic was administered.

In all the following experiments the strength of the arsenious acid solution was in the proportion of twelve grains in one fluid ounce of water.

*Experiment* 16*th; weight* 25 *lbs.*—Six grains administered. Symptoms of poisoning came on in eight minutes; the antidote was then administered. The animal vomited in about five minutes after the administration of the antidote, and appeared to suffer some pain. The vomiting was repeated several times, after which the symptoms of pain gradually subsided and the animal recovered.

*Experiment* 17*th; weight* 18 *lbs.*—Six grains administered, followed immediately by the antidote. Vomiting commenced in about ten minutes, and was repeated two or three times; after this the animal showed no sign of distress.

*Experiment* 18*th; weight* 18 *lbs.*—Six grains of arsenious acid in solution were mixed with about fifteen times its weight of the hydrated sesquioxide of iron and allowed to stand twenty minutes. The mixture was then administered. The closest observation failed to detect any symptom whatever as an effect of the mixture.

*Experiment* 19*th; weight* 15 *lbs.*—Seven grains administered, followed immediately by the antidote. Symptoms appeared in about eight minutes, vomiting in fifteen minutes. The vomiting was repeated three or four times, after which the animal soon recovered, not having showed much signs of suffering.

*Experiment* 20*th; weight* 25 *lbs.*—Seven grains administered. The animal exhibited some signs of pain in about five minutes. A large amount of the antidote was then administered; this was followed by vomiting, which was repeated several times, each vomit showing the presence of the antidote. The animal appeared a little stupid, but showed no signs of pain, but recovered in about five hours.

*Experiment 21st; weight 20 lbs.*—Eight grains administered, followed immediately by the antidote. Symptoms of pain and uneasiness came on in eight minutes, followed by vomiting, which continued about an hour and then ceased. After this the animal showed some signs of pain but recovered in about seven hours.

*Experiment 22d; weight 30 lbs.*—Eight grains administered. Symptoms came on in five minutes; the antidote was administered in three minutes after the first appearance of the symptoms; vomiting came on in less than two minutes after the administration of the antidote. The vomiting continued with an apparent increase of all the other symptoms. The antidote was repeated; after which the animal vomited twice or three times. The symptoms gradually diminished, and the animal recovered.

As a deduction from the above experiments it is obvious, when the cases in which no antidote was given, are compared with those in which it was administered, that the hydrated sesquioxide of iron is an antidote for arsenious acid when administered in solution.—*Med. & Surg. Journal.*

---

Perchloride of Iron.—This powerful styptic and astringent has been heretofore, we believe, in this country, used mainly as an external application. We have heard in one instance of its successful use internally in a case of obstinate nasal hemorrhage. We transcribe below two cases, where it proved valuable as an internal remedy, from the *British and Foreign Med. Chir. Review:*—

Dr. Sassier, of Châlon-sur-Saône, was called to see a man aged 70, who had been seized suddenly with depression, nausea, and shiverings, and three days after these preliminary symptoms there followed epistaxis, hæmatemesis, and hæmaturia; the patient lost blood both by the gums and the rectum. At the same time petechiæ and ecchymoses were developed on the trunk and limbs. Iced drinks were ordered, together with dilute sulphuric acid, and extract of rhatany, but without success, and indeed the symptoms seemed to be increased. The hemorrhage continued, the tongue became dry and black, and the prostration was extreme. Dr. Sassier then prescribed the perchloride of iron, dissolved in distilled water, and sweetened with syrup, to be taken in spoonfuls every hour. The next day the patient's state was the same, but on the succeeding day there was a sensible diminution of the hemorrhage, which ceased on the third day, but the perchloride was continued for two days longer. The disease seemed to be cured, but a week afterwards the hemorrhage reappeared, and the perchloride was again ordered, and after it had been employed two days the bleeding entirely ceased, and was never again renewed. The patient recovered after a prolonged convalescence.

Dr. Bertet relates another very severe case of purpura hæmorrhagica treated successfully by the perchloride of iron, and in this case the remedy was employed to the exclusion of all other medicinal agents. Dr. Bertet considers that at present the perchloride of iron is the best remedy for purpura hæmorrhagica, and that in some cases it is almost infallible.

